# Sublingual dermoid cyst in an infant: A case report and review of the literature

**DOI:** 10.1002/ccr3.2823

**Published:** 2020-08-01

**Authors:** Madeleine Edith Vélez‐Cruz, José Francisco Gómez‐Clavel, Carlos Juan Licéaga‐Escalera, Luis Alberto Montoya Pérez, Juan José Trujillo Fandiño, Cynthia Georgina Trejo Iriarte, Maria Fernanda Ramírez‐Cano, Alejandro García‐Muñoz

**Affiliations:** ^1^ Servicio de Cirugía Maxilofacial Hospital Juárez de México Ciudad de México México; ^2^ Laboratorio de Investigación en Educación y Odontología Carrera de Cirujano Dentista FES‐Iztacala Universidad Nacional Autónoma de México Estado de México México; ^3^ Carrera de Cirujano dentista Laboratorio de Investigación en Odontología Almaraz FES‐Iztacala Universidad Nacional Autónoma de México Estado de México México

**Keywords:** approach, dermoid cyst, epidemiology, infant, treatment

## Abstract

Dermoid cysts usually occur later in the second decade of life; we present the approach of an unusual case of an infant who presented a cyst within the oral cavity, which is important because it can be confused with other pathologies.

## Graphical abstract

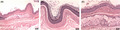

## 1 INTRODUCTION

Dermoid cysts are uncommon benign congenital tumors with an epithelial layer of ectodermal origin that can occur in any region of the body.^1^ Dermoid cysts in the head and neck can be associated with displaced epithelium or entrapped epithelial rest during the midline fusion between the first and second branchial arches that happens during the four weeks of development.^2^, ^3^ The sublingual dermoid cyst is located in the midline, above, or below the mylohyoid muscle.^4^

Dermoid cysts generally present slow and progressive growth, and even if they are congenital, the diagnosis is commonly possible in the second or third decade of life.^5^ About 7% of all dermoid cysts found in the body are formed in the head or neck region, and only 1.6% may occur within the oral cavity.^6^ Another study reported that, out of 1007 tumors in children are on the head and neck, 95 (9.4%) were dermoid cysts and only 3 (0.3%) occurred within the oral cavity.^7^ The treatment of this type of dermoid cysts on the floor of the mouth is surgical; the approach can be either intraoral or extraoral, depending on the location and size of the mass.^8^ A review of the literature from 2000 to 2019 revealed 60 (70) reported cases within the oral cavity dermoid cysts.

The aim of this work is to present a clinical case that shows clinical features, approaches, and follow‐ups as well as the results of a literature review.

## 2 REPORT OF A CASE

**FIGURE 1:**
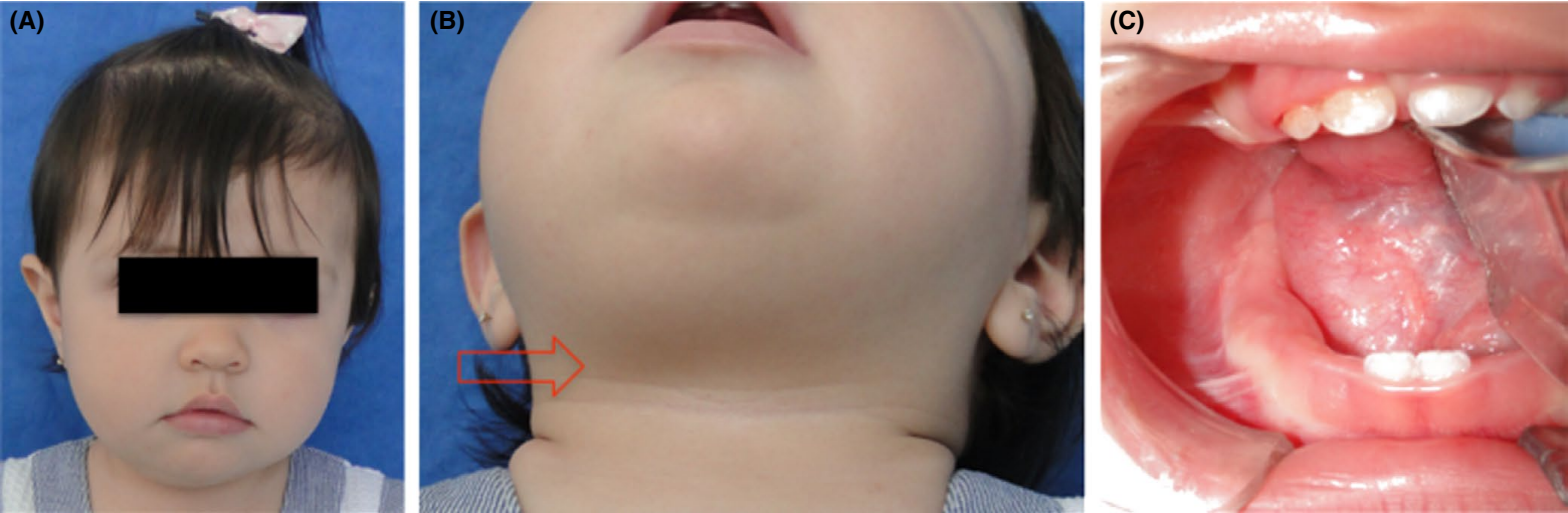
Clinical pictures of the treated patient. A, A picture of the face shows the increase of volume around submandibular region. B, The extraoral region of submandibular area on the right side where there are soft depression and increment in volume. C, Intraoral image shows a volume increase of the floor of the mouth in the right side which is occupied by the lesion with a same color of the adjacent tissue.

In December 2008, a 13‐month‐old female patient was referred to the Oral and Maxillofacial Surgery Department in México City (Hospital Juárez de México) for evaluation of a sublingual mass. It was referred from the pediatric service because she presented swallowing difficulties and an increased volume in the right submandibular region and on the floor of mouth with seven months of evolution (Figure 1A). On clinical examination, apparently there was no pain to palpation, the affected region, and it was movable and the consistency was soft (Figure 1B,C).

**FIGURE 2:**
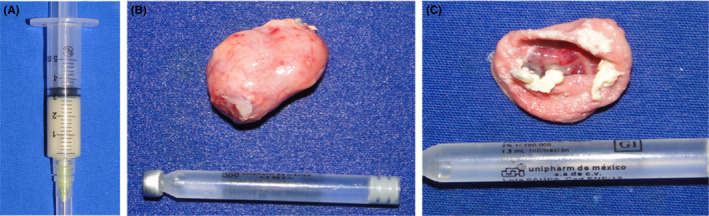
Macroscopic characteristics of the specimen. A, In the exploring procedure, an aspiration was made where we see a large amount of keratin. B, It is the picture shows the integrity and size of the excised specimen (25 × 20 × 10 mm). C, In the sample, keratin mist was exposed as well as the thickness of the capsule.

An ultrasound was requested, in which we identified the right submandibular gland and adjacent to it, we observed an ovoid lesion, moving upwards and laterally with well‐defined edges and a thin wall. The interior showed heterogenicity with multiple small points. Afterward, we then requested the Doppler mode to assess the blood flow and showed abnormal vascular flow in the lesion. Later, an aspiration was performed, obtaining abundant material with cheese‐type appearance (Figure 2A).

**FIGURE 3:**
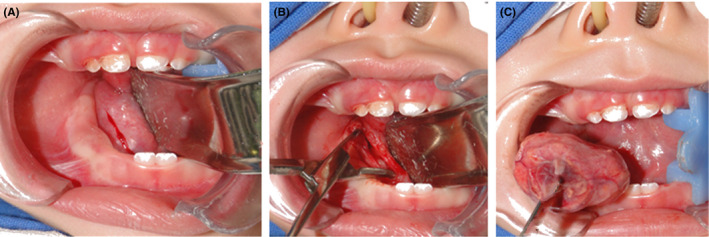
A surgical approach. A, The surgical incision was made by following the path of the submandibular duct. B, Accessing to lesion was made using a roma dissection. C, After performing the surgical procedure, the sample was obtained entirety by including the capsule

The surgical excision was made under general anesthesia with nasal intubation, while using an incision on the floor of the mouth (Figure 3) and close to the frenulum and parallel to the alveolar ridge of approximately two centimeters in length. A blunt dissection was performed, exposing the integrity of the cyst capsule, which was feebly attached to adjacent tissue (Figure 3B), obtaining a single and irregular ovoid lesion of 25 × 20 × 10 mm (Figure 3C), comprising a thick capsule with abundant content with cheese‐type appearance.

**FIGURE 4:**
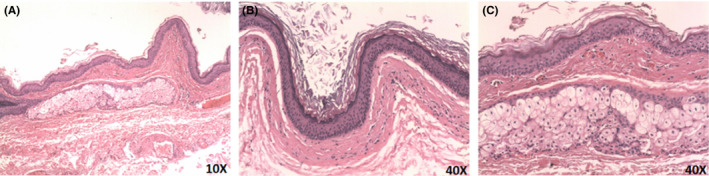
Histological images of dermoid cyst. A, Microscopically, the cyst lining is composed of squamous epithelium with keratin debris without evidence of malignancy and accessory sebaceous gland. B, Amplified vision of the classic fibrous capsule covered by stratified epithelial. C, Amplified vision of the accessory sebaceous gland

**FIGURE 5:**
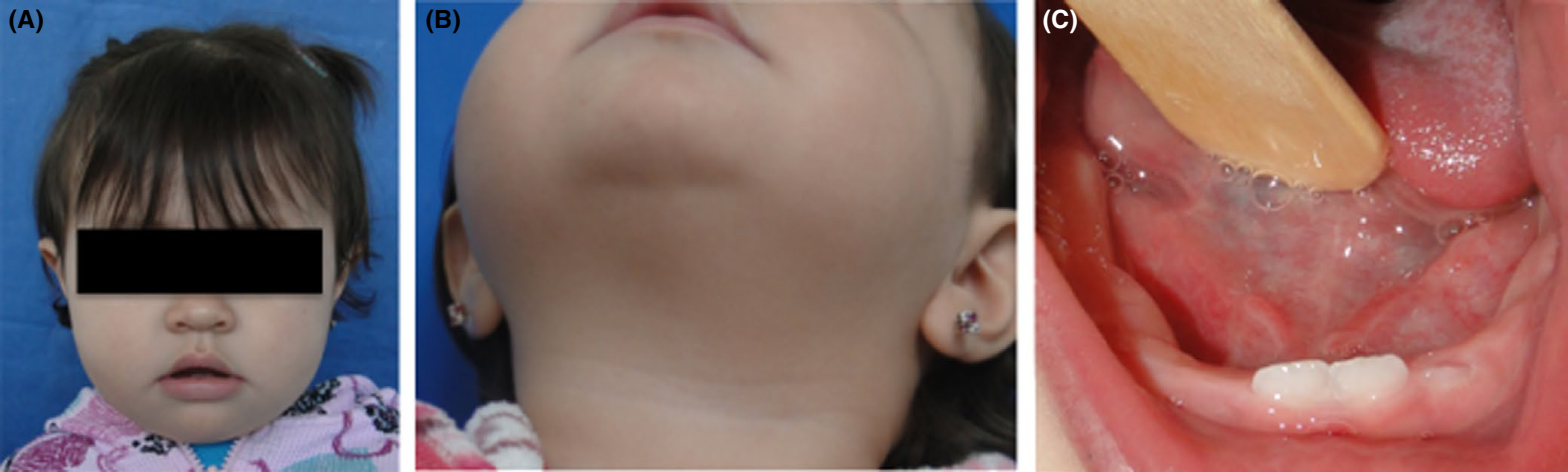
Postoperative control. A, Face of patient, where some pathological characteristics were not perceived. B, Neck and extraoral region shows a normal anatomy. C, The floor of mouth is health, and we could see the preservation of submandibular canal

The histological examination confirmed a diagnosis of dermoid cysts (Figure 4). The cyst contents consisted of lamellated layers of keratin desquamated by the epithelial lining (Figure 4A). Epidermal appendages and connective tissue elements are observed in the wall of the epithelium‐lined cyst (Figure 4B). Finally, clinical examination 1 year after the surgery clinical examination showed no evidence of a disease and the patient was no longer required follow‐up attention (Figure 5).

## 3 LITERATURE REVIEW

An initial search was conducted by PubMed and Google Scholar through 2000 to 2019, based on a combination of the following medical subject headings: Dermoid, Cyst, Floor of the mouth. The inclusion criteria for the election of the reported cases were that they contain a histological study report which described the characteristics of the dermoid cysts (cystic lesion with stratified keratinized epithelium surrounded by a capsule of connective tissue containing skin appendages). The search produced a total of 52 articles, the patient´s ages when the lesion on the floor was detected were from newborn to 59 years old. When we categorized the patient´s ages, it is most often presented in the first three decades of life (Table 1).

**TABLE 1:** Dermoid cysts cases reported from 2000 to 2019

Authors	Age	Frequency	%
Ho^9^; Hemaraju^3^; Anguita^24^; Pan^25^; Schwanke^16^; Gordon^26^ ^;^ Puricelli^19^; Tekkeşİn^27^; Berbel^28^; Naina^29^	0‐9	12	20
Lima^30^; Ege^31^; Seah^23^; Teszler^32^; Ikeda^33^; Montoro^34^; Portelles & Torres^35^; Makos^36^; Metz^14^; Jain^37^; Schwanke^16^; Dillon^11^; Kyriakidou^38^; Patel^39^; Derin^40^; Giarraputo^41^	10‐19	17	28.33
Santos‐Britz^42^; Kutuya^43^; Longo^5^; Tuz^44^; Liceaga^45^; Burger^46^; El‐Hakim^47^; Jadwani^48^; Makos^35^; Ohta^8^; Antunes^49^; Vieira^50^; Sahoo^51^; Mumtaz & Singh^15^; Kumar^52^	20‐29	16	26.66
Fuchshuber^53^; Santos^54^; Aydın^10^; Armstrong^13^; Pirgousis & Fernades^55^; Sun^56^	30‐39	7	11.66
Longo^5^; Durr^57^	40‐49	2	3.33
Vargas^58^; Otonari‐Yamamoto^59^; Devine & Jones^21^; Ariyoshi & Shimahara,^12^ ^;^Lin^1^; Gordon^26^ ^n^	50‐59	6	10
	Total	60	100

The sex prevalence shows us that it presents with no significant differences in both sexes (chi‐square statistic 1.724, *P*‐value: .189, *P* < .05), 40% in females and 60% in males. But, after 30 years, the highest incidence is among men, 84.61%, and only 15.38% among women (Table 2).

**TABLE 2:** Incidence of the dermoid cysts

Age range	Male	Female	Total
0‐60	36	24	60
0‐9	7	5	12
10‐19	8	9	17
20‐29	9	7	16
30‐39	6	1	7
40‐49	2	0	2
50‐59	4	2	6

According to the largest dimension of the lesions reported, the smallest was 1.2 cm^9^ and the largest was 12 cm in its major axis.^10^

In some diagnostic images, the dermoid cysts are observed as sacs with round objects.^1^, ^11^, ^12^

In 58 cases of dermoid cysts of the floor of the mouth, only 55 describe the surgical approach; of these 35 (59.3%), the cyst was enucleated through intraoral approach; and 18 cysts were (30.5%) eliminated by an extraoral approach. In two cases, it was used as a mixed approach^13^, ^14^ and in one case, marsupialization was used.^15^ (Table 3).

**TABLE 3:** Surgical approach

Extraoral enucleation	19
Oral enucleation	35
Extraoral/intraoral enucleation	2
Marsupialization	1
Undetermined	3

## 4 DISCUSSION

The dermoid cysts of the floor on the mouth are rare lesions that originate more frequently in the first decades of life, and the case we present was detected and treated surgically in a female infant of 13 months. Schwanke et al^16^ in 2013 reported a case that was diagnosed and treated in a one‐year‐old male infant. Bloom et al^17^ in 2002 detected a compressible dermoid cyst in a newborn male that was removed at three months. Finally, Hemaraju  et al ^3^ in 2004 tried a 2‐year‐old baby.

The differential diagnosis is made with ranula, mucocele, and Warthon duct obstruction, as well as congenital malformations of the floor of the mouth, such as hemangiolymphangiomas, thyroglossal duct cysts, branchial cysts, and ectopic thyroid tissue.^18^ Puricelli et al^19^ in 2017 reported the case, which had originally being diagnosed as ranula and subjected to two occasions with marsupialization, while Kim et al^20^ in 2006 reported the case of a 3‐month infant with large painless swelling on the floor of the mouth that was treated with marsupialization of the lesion and that nine months later reappeared with high fever because of secondary infection and a swelling on the sublingual and submental area, which was treated with submental and intraoral approach.

Otherwise, Seah^21^ in 2004 reported the case of dermoid cysts within the oral cavity that became infected and that did not respond to antibiotic treatment and that it proceeded with bilateral lymphadenopathy.

In the case presented here, the lesion was diagnosed by ultrasonography and fine canula aspiration with the initial diagnosis of development cysts, which was confirmed by the histopathological study that also defined the type of cyst which according to the presence of sebaceous glands in the connective tissue capsule was diagnosed as a dermoid cyst, and the treatment employed in this case was an intraoral approach because it was above the geniohyoid muscle and an easy access. Some authors have mentioned that an intraoral approach is ideal because it gives a good view of the cyst, an easy access as well as esthetic results, even when the cyst is above the geniohyoid muscle.^22^ Two of the most voluminous lesions reported were enucleated intraorally.^10^

## CONFLICT OF INTEREST

None declared.

## AUTHORS’ CONTRIBUTIONS

MEVC, CGLE, LAMP, and JJTF: examined and treated the patient, and wrote and edited the manuscript; MEVC and AGM: conceived and lead the idea for the case report; JFGC, CGTI, AGM, and MFRC: contributed to the design and editing the manuscript.
